# Time Course of the Development of Nonalcoholic Fatty Liver Disease in the Otsuka Long-Evans Tokushima Fatty Rat

**DOI:** 10.1155/2013/342648

**Published:** 2013-05-12

**Authors:** Yi-Sun Song, Cheng-Hu Fang, Byung-Im So, Jun-Young Park, Yonggu Lee, Jeong Hun Shin, Dae Won Jun, Hyuck Kim, Kyung-Soo Kim

**Affiliations:** ^1^Graduate School of Biomedical Science and Engineering, Hanyang University, Seoul 133-792, Republic of Korea; ^2^Department of Internal Medicine, Hanyang University College of Medicine, 17 Haengdang-dong, Sungdong-ku, Seoul 133-792, Republic of Korea; ^3^Department of Internal Medicine, College of Medicine, Yanbian University, Yanji 133000, China; ^4^Department of Thoracic and Cardiovascular Surgery, Hanyang University College of Medicine, Seoul 133-792, Republic of Korea

## Abstract

Nonalcoholic fatty liver disease (NAFLD) is considered a hepatic manifestation of metabolic syndrome. In this study, we investigated histological and biochemical changes in NAFLD and the gene expression involving *de novo* lipogenesis in Otsuka Long-Evans Tokushima fatty (OLETF) rats. We used OLETF rats and Long-Evans Tokushima Otsuka (LETO) rats as animal models of NAFLD and as controls, respectively. Consistent observations were made at 4-week intervals up to 50 weeks of age, and all rats were fed ad libitum with standard food. Biochemical and histological changes were observed, and gene expression involved in *de novo* lipogenesis was measured using real-time polymerase chain reactions. As a results hepatic micro- and macrovesicular steatosis and hepatocyte ballooning were evident in the OLETF rats at 22–38 weeks of age but disappeared after 42 weeks; no fibrosis or collagen deposition was observed. Gene expression involved in *de novo* lipogenesis followed a pattern similar to that of the histological changes. In conclusion, in the absence of dietary manipulation, hepatic steatosis in OLETF rats is evident at 22–38 weeks and declines after 42 weeks. Therefore, OLETF rats at 22–38 weeks are recommended as animal models of hepatic steatosis.

## 1. Introduction

Nonalcoholic fatty liver disease (NAFLD) is presently well recognized as a hepatic manifestation of metabolic syndrome [1]. NAFLD is strongly associated with obesity, type 2 diabetes mellitus, and hyperlipidemia [2]. The spectrum of NAFLD ranges from simple steatosis thorough nonalcoholic steatohepatitis (NASH) to cirrhosis [3]. Approximately 20%–30% of NAFLD patients progress to NASH and to cirrhosis [4]. NAFLD is estimated to affect up to 30% of all adults in the United States and up to 70% of obese individuals [5]. Day and James have proposed a two-hit hypothesis to explain the progression of NAFLD. The first hit involves the deposition of triglycerides in hepatocytes (hepatic steatosis) and the second hit refers to the cellular events leading to hepatic inflammation (NASH) [6].

Many NAFLD studies have used animal models such as the rat model of methionine and choline deficiency- (MCD-) induced NASH [7], high fat diet-induced hepatic steatosis [8], and fructose-induced NAFLD [9]. Mutant animals such as db/db mice [10], ob/ob mice [11], and Otsuka Long-Evans Tokushima fatty (OLETF) rats have also been used as models of NAFLD [12].

 The OLETF rat is a well-established model of metabolic syndrome, characterized by abdominal obesity, insulin resistance, hypertension, and hyperlipidemia [13, 14]. The cholecystokinin- (CCK-) 1 receptor is not made in OLETF rats because of a genetic deletion [15, 16], and this absence of the CCK-1 receptor—one of the most abundant neurotransmitter peptides in the brain—may lead to metabolic syndrome because of a lack of satiety [17]. The OLETF rats originated from an outbred colony of the Long-Evans rat strain. A control strain, the LETO rat, which originated from the same colony, makes the CCK-A receptor and is diabetes resistant [16]. One of the advantages of the OLETF rat is that it develops NAFLD spontaneously in the absence of extreme dietary manipulation [18]. In addition, its natural pattern of progression closely resembles that observed in obese humans [19].

In previous studies, OLETF rats at 32 weeks of age were used to investigate the effect of rosiglitazone on hepatic steatosis [20]. Yeon et al. observed hepatic steatosis in 40-week-old OLETF rats, whereas 12- and 28-week olds did not show histological changes [21]. Also, Seo et al. used the OLETF rat at 12 weeks of age as a NAFLD model [22], and Borengasser et al. similarly used 13-week-old OLETF rats [23]. Guo et al. observed hepatic steatosis and vacuolization in the OLETF rat at 16 weeks of age, and this gradually worsened to 30 weeks of age [24]. For use as an animal model of NASH, OLETF rats are fed an MCD diet for 8 weeks from 24 weeks of age [25]. The OLETF rat has been used as an animal model for NAFLD in many experiments; however, the time course of histological and biochemical changes in OLETF rats has not been clearly defined. Determining the therapeutic effects of drugs is difficult because of a lack of information about the development of disease in this animal model.

The aim of this study was to establish the time course of the development of NAFLD in the OLETF rat model. We therefore investigated the histological and biochemical changes associated with NAFLD in OLETF rats up to 50 weeks of age, as well as expression of genes involved in *de novo* lipogenesis. 

## 2. Materials and Methods

### 2.1. Animals

 This study was performed in compliance with the ARRIVE guidelines for research [26], and the Hanyang University Institutional Animal Care and Use Committee approved all protocols. Four-week-old OLETF rats (*n* = 40) and control Long-Evans Tokushima Otsuka (LETO) rats (*n* = 5) were supplied by the Tokushima Research Institute, Otsuka Pharmaceutical Co. (Tokushima, Japan). All animals were provided with standard rodent chow ad libitum (20.14% protein, 13.12% moisture, 5.9% fat, and 5.02% fiber; Lab Rodent Chow; 38057; Purina Korea Inc., Republic of Korea). They were housed under conditions of controlled temperature (23 ± 2°C) and humidity (55 ± 5%) with a 12 h artificial light/dark cycle in a specific pathogen-free facility of the Hanyang University Medical School Animal Experiment Center.

### 2.2. Experimental Protocols

The experiment was initiated when the rats were 10 weeks of age. OLETF rats were killed for histological analysis at 4-week intervals up to 50 weeks of age, while LETO rats were killed only at 50 weeks of age. All rats were fasted for 12 h prior to being killed, and blood was collected from tail veins. Body weights were measured at 4-week intervals. 

### 2.3. Biochemical Analysis

Serum was obtained from blood by centrifugation and stored at −70°C. Serum glucose, total cholesterol (TC), triglyceride (TG), insulin, alanine aminotransferase (ALT), aspartate aminotransferase (AST), and free fatty acid (FFA) levels were measured using an autoanalyzer (Olympus GmbH, Germany) [27, 28]. Serum glucose, TC, and TG levels were measured at 4-week intervals up to 50 weeks of age, whereas serum insulin, ALT, AST, and FFA levels were measured only at 50 weeks of age. Insulin resistance was estimated by homeostasis model assessment of insulin resistance (HOMA-IR) using the following formula: HOMA-IR = fasting insulin (*μ*U/mL) × fasting plasma glucose (mmol/l)/22.5 [29].

### 2.4. Histological Staining and Grading

Livers were fixed in 10% buffered formalin and embedded in paraffin. The severity of histological changes was assessed by hematoxylin and eosin (H&E) and Masson's trichrome (MT) staining. Oil Red O staining was performed as previously described [12]. Three regions of digitized images of the Oil Red O-stained liver sections from each animal were selected at random from the individual sections and were quantified using the Leica image analysis system [30, 31]. The stained sections were photographed using a light microscope (Leica DM 4000B, Germany). 

### 2.5. RNA Isolation

RNA was isolated from 40 mg samples of liver tissue using Qiazol reagent (QIAgen, USA). RNA concentration was measured with a NanoDrop ND-2000 UV/Vis spectrophotometer (Thermo Fisher Scientific Inc., USA), and RNA purity was determined by measuring the ratio of absorbance at 260 and 280 nm, which ranged from 1.8 to 2.0.

### 2.6. Quantitative Real-Time Polymerase Chain Reaction (PCR)

Total RNA was extracted from 20 mg samples of liver tissue using Qiazol reagent (QIAgen, Valencia, CA) following the manufacturer's instructions. Complementary DNA (cDNA) was synthesized from 3 *μ*g of RNA using Moloney Murine Leukemia virus reverse transcriptase primed with oligo(dT) (Invitrogen, Carlsbad, USA). mRNA expression was quantified by real-time PCR (Roche, Basel, Switzerland) using a LightCycler FastStart DNA Master SYBR Green I kit (Roche Diagnostics, IN, USA). The following real-time PCR amplification protocol was used: incubation for 10 min at 95°C followed by 45 cycles of 10 s at 95°C, 10 s at 60°C, and 8 s at 72°C and a final dissociation curve step at 65°C for 15 s. The crossing point (Cp) of each PCR was automatically determined by the LightCycler program. PCR reactions for each sample were run in duplicate. The specific primers for the target genes (sterol regulatory element-binding protein- (SREBP-) 1c, stearoyl-CoA- (SCD-) 1, carbohydrate response element-binding protein (chREBP), fatty acid synthase (FAS), and acetyl-CoA carboxylase (ACC)) and glyceraldehyde-3-phosphate dehydrogenase (GAPDH) are listed in Table 1. The expression levels of target genes were normalized to those of GAPDH. 

### 2.7. Statistical Analysis

All data are presented as means ± SD, except for the Oil Red O staining data obtained with the image analysis system, which are presented as mean ± SE. Comparisons between groups were made using one-way analysis of variance followed by a post hoc Tukey's test using Statistical Program for the Social Sciences (SPSS software version 17.0 (SPSS, Inc., USA)). Side-to-side comparisons within the same group were made using Student's *t*-test for paired data. Values of *P* < 0.05 were considered statistically significant.

## 3. Results

### 3.1. Animal Characteristics and Biochemical Analysis

**Figure 1:**
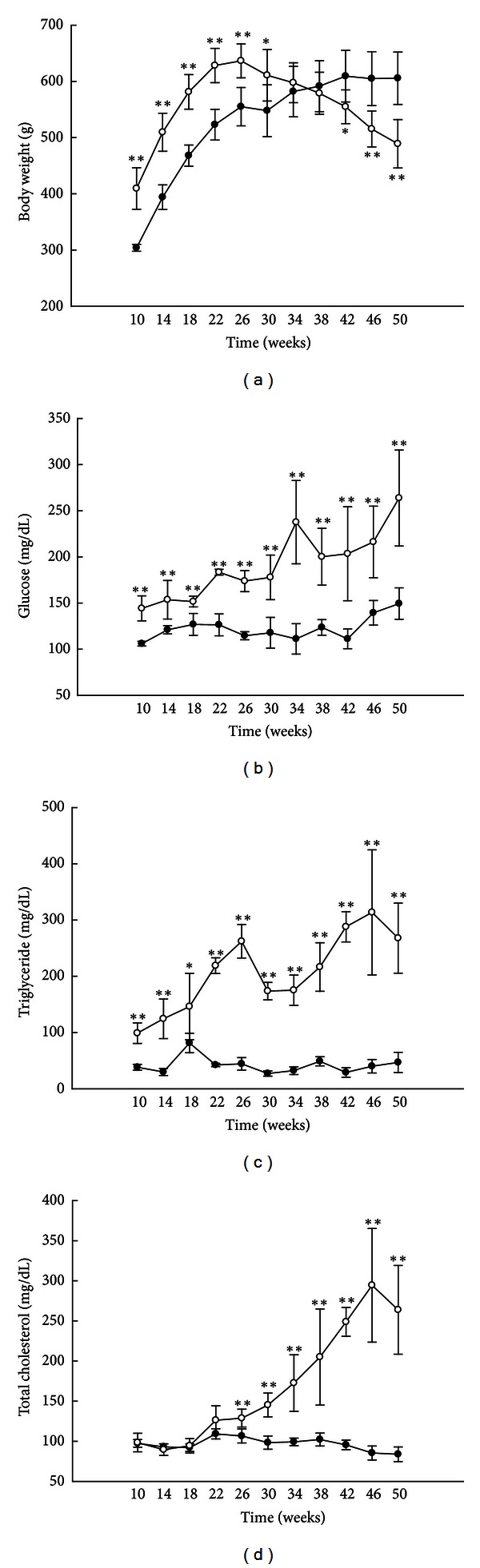
Animal characteristics and biochemical analyses. Body weights ((a), *n* = 6) and serum glucose ((b), *n* = 5), triglyceride ((c), *n* = 5), and total cholesterol ((d), *n* = 5) levels. White circles: OLETF rats; black circles: LETO rats. All data are expressed as means ± SD. *P < 0.05, **P < 0.01 compared with LETO rats.

Body weight was significantly higher in the OLETF rats than in the LETO rats until 30 weeks of age and fell below that of the LETO rats by 42 weeks (Figure 1(a)). Serum glucose and TG levels were higher in the OLETF rats than in the LETO rats at all ages (Figures 1(b) and 1(c)), and TC levels were higher in the OLETF rats than in the LETO rats after 26 weeks (Figure 1(d)). At 50 weeks, levels of AST and free fatty acid were higher in the OLETF rats than in the LETO rats, but no significant differences were observed in insulin and ALT levels (Table 2). In the OLETF rats, levels of HOMA were lower at 50 weeks of age than at 30 weeks of age (Table 3).

### 3.2. Liver Histology

**Figure 2:**
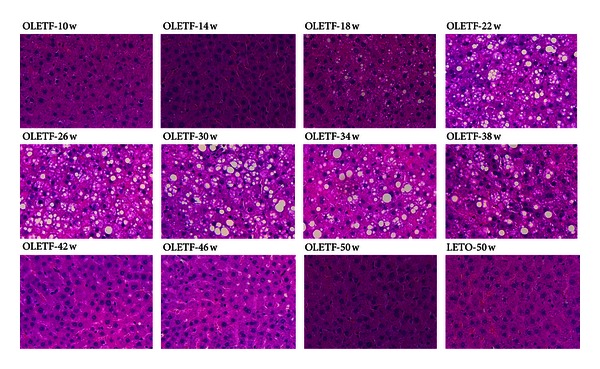
Liver histology by hematoxylin and eosin (H&E) staining. Histological changes in liver tissue observed by H&E staining in OLETF rats at 4-week intervals up to 50 weeks of age (magnification, ×200).

**Figure 3:**
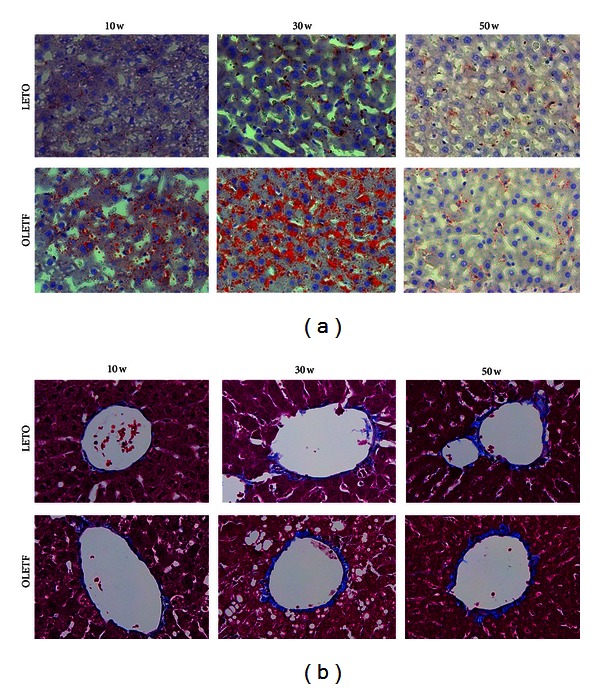
Liver histology. Representative images of Oil Red O (a) and Masson's trichrome (MT) staining (b) of the livers at 10, 30, and 50 weeks of age (magnification, ×400).

The progression of NAFLD was confirmed in the liver tissue of OLETF rats by H&E (4-week intervals), Oil Red O, and MT staining (at 20-week intervals). Microvesicular steatosis was observed in OLETF rats at 18 weeks, and micro- and macrovesicular steatosis and hepatocyte ballooning became evident from 22–38 weeks of age and declined after 42 weeks (Figure 2). The area of lipid droplets, as visualized by Oil Red O staining, was larger in the OLETF rats than in the LETO rats at 10 and 30 weeks of age, but there was no significant difference at 50 weeks (Figure 3(a) and Table 4). In the OLETF rats, no fibrosis or collagen deposition was evident in perivenular regions (i.e., encompassing terminal hepatic veins) by MT staining (Figure 3(b)). 

### 3.3. Expression of Genes Related to Lipogenesis

**Figure 4:**
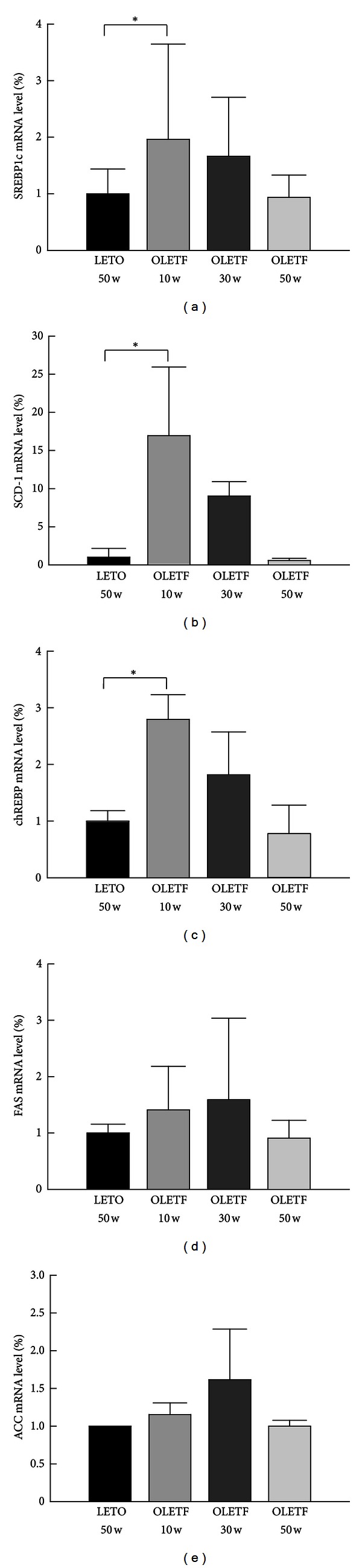
Expression of genes related to lipogenesis. Levels of SREBP-1c, SCD-1, chREBP, FAS, and ACC mRNA in the liver at 10, 30, and 50 weeks of age. ((a)–(e)) Sterol regulatory element-binding protein (SREBP) 1c, stearoyl-CoA- (SCD-) 1, carbohydrate response element-binding protein (chREBP), fatty acid synthase (FAS), and acetyl-CoA carboxylase (ACC) mRNA levels, respectively. Mean values were obtained from livers of 3 separate animals. Polymerase chain reactions performed in duplicate. The transcript levels normalized relative to GAPDH expression. Data are expressed as means ± SD. *P < 0.05 compared with LETO rats.

Levels of SREBP-1c, SCD-1, chREBP, FAS, and ACC mRNA in the liver of rats were measured by real-time PCR (OLETF, at 20-week intervals; LETO, at 50 weeks of age) (Figure 4). Levels of SREBP-1c, SCD-1, and chREBP mRNA were higher at 10 weeks of age in the OLETF rats than in the LETO rats (Figures 4(a)–4(c)), but levels of FAS and ACC mRNA did not differ significantly at any time point (Figures 4(d) and 4(e)). 

## 4. Discussion

In this study, we examined the development with age of NAFLD in OLETF rats. We demonstrated that hepatic steatosis was evident in OLETF rats in the absence of dietary manipulation between 22 and 38 weeks of age and declined after 42 weeks. Fibrosis and collagen deposition in perivenular regions did not appear within 50 weeks. Further, the expression of genes related to *de novo* lipogenesis increased at 10 and 30 weeks in OLETF rats and declined by 50 weeks. 

OLETF rats spontaneously develop insulin resistance, type 2 diabetes [32], metabolic syndrome [17], and hepatic steatosis [21]. The OLETF rat is an established model of NAFLD because it follows a pattern of development similar to that of the human disease and it occurs in the absence of extreme dietary manipulation [19, 33]. 

We found that OLETF rats had higher serum levels of glucose, triglyceride, and total cholesterol than LETO rats (Figures 1(b) and 1(c)). However, their body weight started declining at 30 weeks of age and, by 42 weeks, was significantly lower than that of the LETO rats (Figure 1(a)). In previous studies, the body weight of OLETF rats also peaked between 25 and 30 weeks [5, 34, 35]. Hence, the decline in body weight after 30 weeks may be a characteristic of OLETF rats. The HOMA scores of the OLETF rats were also lower at 50 weeks than at 30 weeks (Table 3).

As expected, hepatic micro- and macrovesicular steatosis and hepatocyte ballooning were not observed in the OLETF rats before 14 weeks of age; they appeared at 18 weeks and were very evident between 22 and 38 weeks. Surprisingly, however, they disappeared by 42 weeks (Figure 2). The area of lipid droplets also peaked at approximately 30 weeks of age (Figure 3(a)). In this study, hepatic steatosis in OLETF rats peaked at approximately 30 weeks and disappeared by 42 weeks in conjunction with the reduction insulin resistance and SREBP-1c expression. Also, previous studies have demonstrated that insulin resistance is closely related to SREBP-1c levels, and the reduction of SREBP-1c levels could contribute to the improvement of insulin resistance [36]. We speculate that reduction of hepatic steatosis in OLETF rats is related to the decline in insulin resistance and SREBP-1c expression. However, further study is required to confirm these mechanisms. 

Fibrosis and collagen deposition in the perivenular regions of the livers of OLETF rats did not increase over time (Figure 3(b)). This indicates that OLETF rats are not useful as models of NASH in the absence of dietary manipulation.

We showed that the expression of genes related to *de novo* lipogenesis in OLETF rats increased at 10 and 30 weeks of age and then declined by 50 weeks. Previous studies have shown that hepatic lipogenesis increases in NAFLD [37]. Further, inhibition of SREBP-1c, a master regulator of genes involving hepatic lipogenesis, reduces the expression of ACC, FAS, and SCD-1 [38]. These findings may account for why 50-week-old OLETF rats are not appropriate models of NAFLD. To clarify the situation, however, studies of other pathways such as fatty acid oxidation in mitochondria and peroxisomes and fatty acid transport in OLETF rat during the development of NAFLD are needed.

This study has several limitations. First, the mechanism responsible for the reduction of hepatic steatosis after 42 weeks of age in the OLETF rats is unclear. Second, our study involved only a small number of animals. Finally, time course data of food intake in the OLETF rats is limited. However, we suppose that the body weight loss in the OLETF rats is not due to reduction of food intake, because previous studies have shown that the food intake of OLETF rat does not decline until at least 38 weeks of age [39].

In summary, we have demonstrated that hepatic steatosis in OLETF rats in the absence of dietary manipulation increases from 22–38 weeks of age and declines after 42 weeks. We recommend the use of OLETF rats at 22–38 weeks of age as animal models of hepatic steatosis, but these rats are not useful as models of NASH in the absence of dietary manipulation. Our findings provide insight into the use of OLETF rats as models of NAFLD and define the optimal time for observing hepatic steatosis. We believe these data will contribute to the study of NAFLD using OLETF rats.

## Figures and Tables

**Table 1:** Sequences of primers.

Primer	Sequences (5′ to 3′)	Size (bp)
SREBP-1c	GCT ACC GTT CCT CTA TCA ATG ACA A	81
	CAG ATT TAT TCA GCT TTG CCT CAG T	
SCD-1	TTC TTG AGA TAC ACT CTG GTG CTC A	97
	GAG ATT GAA TGT TCT TGT CGT AGG G	
chREBP	CAG TAT GTG GCT TCG TAA CTC CTC T	89
	CCA GTA ATT ACC CTC CAA GAC AAC A	
FAS	TCC ACA GCT CTT ACA GTG AGA ATC A	99
	CTT CTC CAG GGT GGG GAC CAG	
ACC	AGA GTG AGT GCT CTC AAT TCT GTC C	97
	GTC CTT CTT CTT TCC CGA TAA TGT C	
GAPDH	CCT TCT CTT GTG ACA AAG TGG ACA T	96
	CGT GGG TAG AGT CAT ACT GGA ACA T	

SREBP: sterol regulatory element-binding protein; SCD: stearoyl-CoA; chREBP: carbohydrate response element-binding protein; FAS: fatty acid synthase; ACC: acetyl-CoA carboxylase; GAPDH: glyceraldehyde-3-phosphate dehydrogenase.

**Table 2:** Biochemical analyses of serum obtained from the LETO and OLETF rats at 50 weeks of age.

	LETO	OLETF
Insulin (uIU/mL)	6.16 ± 1.61	6.32 ± 0.39
Alanine aminotransferase (U/L)	44.20 ± 4.66	43.60 ± 9.02
Aspartate aminotransferase (U/L)	74.20 ± 14.34	122.20 ± 8.23**
Free fatty acid (*µ*Eq/L)	712.00 ± 25.11	840.80 ± 83.05*

OLETF: Otsuka Long-Evans Tokushima fatty rats; LETO: Long-Evans Tokushima rats. All data are expressed as means ± SE. *P < 0.05, **P < 0.01 compared with LETO rats (*n* = 5).

**Table 3:** HOMA-IR level of the LETO and OLETF rats at 30 and 50 weeks of age.

	30 weeks of age	50 weeks of ages
LETO	14.74 ± 6.73	10.70 ± 2.46
OLETF	27.87 ± 5.60	15.20 ± 1.12*

HOMA-IR: homeostasis model assessment of insulin resistance; HOMA-IR = fasting insulin (*μ*U/mL) × fasting plasma glucose (mmoL/L)/22.5. OLETF: Otsuka Long-Evans Tokushima fatty rats; LETO: Long-Evans Tokushima rats. All data are expressed as means ± SE. *P < 0.05 compared with at 30 weeks of age (*n* = 3−5).

**Table 4:** Histological parameter.

	Hepatic Oil Red O (% staining)	
Age (week)	LETO	OLETF
10	0.17 ± 0.07	0.99 ± 0.36*
30	0.44 ± 0.09	17.61 ± 0.81**
50	0.24 ± 0.13	0.36 ± 0.13

OLETF: Otsuka Long-Evans Tokushima fatty rats; LETO: Long-Evans Tokushima rats. All data are expressed as means ± SE. *P < 0.05, **P < 0.01 compared with LETO rats (*n* = 3).

## Abbreviations

NAFLD: Nonalcoholic fatty liver disease
OLETF: Otsuka Long-Evans Tokushima fatty
LETO: Long-Evans Tokushima Otsuka
NASH: Nonalcoholic steatohepatitis
MCD: Methionine and choline deficiency
CCl_4_: Carbon tetrachloride
CCK: Cholecystokinin
SREBP: Sterol regulatory element-binding protein
SCD: Stearoyl-CoA
chREBP: Carbohydrate response element-binding protein
FAS: Fatty acid synthase
ACC: Acetyl-CoA carboxylase
TC: Total cholesterol
TG: Triglyceride
ALT: Alanine aminotransferase
AST: Aspartate aminotransferase
FFA: Free fatty acids
H&E: Hematoxylin and eosin
MT: Masson's trichrome.
